# Mind–body therapies and their interplay with the immune system in children and adolescents: a protocol for a systematic review and meta-analysis

**DOI:** 10.1186/s13643-025-02812-4

**Published:** 2025-04-04

**Authors:** Steven Schepanski, Gonza B Ngoumou, Anna Katharina Koch, Marleen Schröter, Robert Roehle, Georg Seifert

**Affiliations:** 1 Charité – Universitätsmedizin Berlin, Corporate Member of Freie Universität Berlin, Humboldt-Universität zu Berlin, Charité Competence Center for Traditional and Integrative Medicine (CCCTIM), Berlin, Germany; 2 Charité – Universitätsmedizin Berlin, corporate member of Freie Universität Berlin and Humboldt-Universität zu Berlin, Department of Pediatrics, Division of Oncology and Hematology, Berlin, Germany; 3 Charité – Universitätsmedizin Berlin, Corporate Member of Freie Universität Berlin, Humboldt-Universität zu Berlin, Institute for Biometry and Clinical Epidemiology, Berlin, Germany; 4 Charité – Universitätsmedizin Berlin, Corporate Member of Freie Universität Berlin, Humboldt-Universität zu Berlin, Clinical Trial Office, Berlin, Germany; 5 Charité – Universitätsmedizin Berlin, Corporate Member of Freie Universität Berlin, Humboldt-Universität zu Berlin, Berlin Institute of Health, Berlin, Germany

**Keywords:** Mind–body therapies, Children, Adolescents, Inflammation, Immune system, Systematic review, Meta-analysis, Complementary medicine

## Abstract

**Background:**

Chronic inflammation is a critical public health concern that, in children and adolescents, increases the long-term risk of a variety of different health issues. While mind–body therapies like yoga, meditation, and acupuncture have shown promise in modulating immune responses in adults, their safety and effectiveness in pediatric populations remain underexplored. This protocol outlines the methodology for a systematic review and meta-analysis aimed at evaluating the effects of mind–body therapies on immune modulation in children and adolescents.

**Methods:**

This systematic review and meta-analysis will follow PRISMA 2020 guidelines. We will include randomized controlled trials, non-randomized controlled trials, cohort studies, and case–control studies that examine the relationship between mind–body therapies and immune markers in pediatric populations. Electronic searches will be conducted in MEDLINE, Embase, PsycINFO, CINAHL, Web of Science, and the Cochrane Library, supplemented by trial registries. Risk of bias will be assessed using the Cochrane Risk of Bias Tool (RoB 1), the Risk of Bias in Non-randomized Studies of Interventions (ROBINS-I), and the Newcastle–Ottawa Scale (NOS). Two independent reviewers will screen studies, extract data, and assess study quality, with a third reviewer resolving any discrepancies. Results will be synthesized both narratively and through meta-analysis using R software.

**Discussion:**

The review will evaluate the effectiveness and safety of mind–body therapies on immune markers in children and adolescents. The synthesized evidence will guide clinical practice and public health policies in integrating mind–body therapies into pediatric care. The findings will also provide a foundation for future research and policymaking in this area.

**Systematic review registration:**

PROSPERO CRD42024546585.

**Supplementary Information:**

The online version contains supplementary material available at 10.1186/s13643-025-02812-4.

## Background

Chronic inflammation has gained increased recognition over the past decade as a significant public health concern, particularly among children and adolescents [1–4]. Low-grade, chronic inflammatory responses can profoundly influence the health and development of young individuals, contributing to increased morbidity and mortality in adulthood worldwide [5–8]. Chronic inflammation is implicated in the pathogenesis of various health issues, including cardiovascular diseases [9, 10], insulin-resistant syndromes such as type 2 diabetes [11–13], mental [14, 15], and autoimmune diseases [16, 17].

## Introduction

The prevalence of chronic inflammation is increasing, driven by unhealthy lifestyle factors such as poor diet [18], lack of exercise [19], and environmental pollutants [20, 21]. Children and adolescents are particularly vulnerable, as early-life inflammation significantly increases the risk of developing chronic diseases later in life, including autoimmune conditions such as type 1 diabetes [22, 23] and juvenile idiopathic arthritis [24]; cardiovascular diseases such as atherosclerosis [25] and hypertension [26]; and mental conditions such as depression [27] and anxiety [28].

Chronic inflammation disrupts healthy immune regulation, damaging tissues and elevating long-term disease risks [29, 30]. According to the Barker Hypothesis [31], early life conditions, including inflammation, program long-term health outcomes [29]. For example, elevated markers such as C-reactive protein (CRP) and interleukin (IL)−6 have been linked to an increased risk for metabolic syndrome [32, 33], cardiovascular disease [34], and mental disease later in life [35].

Early interventions to prevent chronic inflammation can improve quality of life and reduce the incidence of chronic diseases [1, 36]. Monitoring inflammatory markers such as CRP and the erythrocyte sedimentation rate (ESR) is crucial for prevention and treatment. Interventions such as dietary changes [37], physical activity [38], and stress management [39] effectively modulate immune responses, decreasing chronic inflammation, and its risks. Emerging therapies, including mind–body therapies (MBTs), offer promising avenues for immune modulation [40, 41].

### Mechanisms of MBT influence on immune outcomes

MBTs influence inflammation through multiple mechanisms. By reducing stress, MBTs modulate the hypothalamic–pituitary–adrenal (HPA) axis, decreasing cortisol and systemic inflammation. They also reduce proinflammatory cytokines and enhance anti-inflammatory markers [40, 42]. Additionally, MBTs improve vagal tone, activating the parasympathetic nervous system and suppressing sympathetic-driven inflammation. Emerging evidence suggests that MBTs induce epigenetic changes that sustain immune homeostasis, providing long-term anti-inflammatory effects [43–45].

#### Previous reviews

The latest meta-analysis by Morgan et al. in 2014 [40] included 34 studies with a total of 2219 participants. The analysis revealed that MBT practices, such as Tai Chi, Qi Gong, meditation, and yoga, had a moderate effect on decreasing CRP levels (effect size, 0.58; 95% confidence interval (CI), 0.04–2.12). However, the reduction in IL-6 was smaller and not statistically significant (effect size, 0.35; 95% CI, − 0.04–0.75), and the effect on tumor necrosis factor (TNF)-α was negligible (effect size, 0.21; 95% CI, − 0.15–0.58). For antiviral immune markers, the effect was also minimal, with negligible changes in CD4^+^ T-cell counts, and NK cell counts (effect size, 0.12; 95% confidence interval, − 0.21–0.45). Despite these limited effects on certain markers, the review did suggest that MBTs could enhance immune responses to vaccinations, indicating the potential for immune modulation, which warrants further study. However, a critical limitation of this analysis is the focus on raw cell counts without considering the balance of immune cell populations. The immune system’s function depends not only on the quantity of specific cells but also on the balance between different types of immune cells, which orchestrate pro- and anti-inflammatory responses. Thus, changes in single cell counts alone may not fully reflect the immune modulation induced by MBTs.

An older systematic review [46], which included 111 studies with 4777 participants, focused on various MBTs, such as relaxation training, cognitive-based stress management, and hypnosis. The review revealed that NK cell activity and CD4^+^ T-cell counts were the most studied outcomes. However, the evidence for the effectiveness of these interventions has generally been limited or inconclusive across most categories. Notably, relaxation training has shown the strongest evidence for influencing immune outcomes, particularly through increasing levels of immunoglobulin A (IgA). This review highlighted significant methodological limitations in the literature and called for more rigorous trials to better understand the impact of MBTs on the immune system. This gap is critical, as childhood and adolescence are periods of significant immune system development, and the potential interaction between MBTs and immune responses to vaccinations and other health interventions needs to be better understood.

### Rationale

The role of age has often been overlooked in previous meta-analyses. Immune system function varies with age, influencing responses to infections, inflammation, and therapies. In children, the immune system matures over time, resulting in distinct inflammatory responses compared with adults. Therefore, age-specific research is essential for understanding and managing these risks.

Chronic inflammation is a major public health issue in children and adolescents, increasing the risk of diseases such as type 1 diabetes [22, 23], juvenile idiopathic arthritis [24], hypertension [26], atherosclerosis [25], and mental health disorders such as depression and anxiety [27, 28]. The complex interaction of genetic, environmental, and psychosocial factors makes age-specific research critical.

No previous systematic reviews have focused exclusively on the effects of MBTs in children, hindering conclusions about their effectiveness and safety. Targeted research is needed to develop effective interventions for modulating immune responses in children. The inclusion of non-English language studies, facilitated by artificial intelligence (AI) translation tools, can enrich the evidence base by incorporating diverse cultural perspectives and scientific findings.

This research has practical and clinical implications. Robust evidence on the effectiveness and safety of MBTs for modulating immune markers in children and adolescents can inform public health policy and clinical practice. The findings will help policymakers understand the importance of integrating MBTs into healthcare programs for young populations and developing guidelines for managing chronic inflammation in pediatric patients.

## Methods and design

### Objectives

The overall objective of this systematic review and meta-analysis is to assess the effects of MBTs on immune responses in children and adolescents.

#### Primary objectives

The primary objectives of this review are threefold: First, to evaluate the impact of MBT on proinflammatory markers such as, but not limited to, tumor necrosis factor (TNF)-α, IL-6, chemokines such as CXCL8, and acute phase proteins such as CRP and serum amyloid A. This objective focuses on the extent to which MBTs can reduce *proinflammatory* processes, which are critical factors in many diseases [1]. Second, to determine the effects of MBT on *anti-inflammatory* markers, including but not limited to IL-10 and growth factors such as transforming growth factor (TGF)-β. These markers are of particular interest because they counteract proinflammatory processes and promote healing [47]. Third, the effects of MBT on *dual-function immune markers*, which can be pro- and anti-inflammatory, should be assessed. We consider immune cell populations such as T, B, NK, and mast cells; monocytes; and antibodies against immunoglobulin (Ig)A, IgG, IgM, and other markers such as ferritin, fibrinogen, and ESR. By exploring these markers, this objective aims to provide a comprehensive view of how MBT can balance immune function in children and adolescents.

#### Secondary objectives

The secondary objectives of this systematic review and meta-analysis aim to establish a comprehensive and detailed association between MBT and aspects of the human immune system. A key question we need to answer concerns the heterogeneity of the study participants. For this reason, we want to compare the effect of MBTs in studies with a population consisting exclusively of children and adolescents under 21 years of age with mixed populations of children, adolescents, and adults. This comparison will answer the question of whether the age composition of the studies themselves influences the effectiveness of MBTs.

Another secondary objective is to determine the differential effects of MBTs in healthy children and adolescents compared with those in children with immunological impairments, such as human immunodeficiency virus (HIV)-infected children. This subgroup analysis should provide information on how MBTs work in individuals with different baseline immune statuses and thus provide evidence-based results for generalizing MBTs to disease patterns.

#### Exploratory objectives

In addition, to the primary and secondary objectives, this review will explore broader aspects of MBTs. Beyond immune markers, this study will assess the safety of MBTs in pediatric populations. This includes evaluating adverse effects or risks associated with MBTs, as well as dropout rates, to determine their suitability for integration into pediatric care. Assessing the safety of MBT interventions is a key prerequisite for their wider acceptance and use in clinical practice. Additionally, this review will assess whether the benefits of MBTs on immune function are sustained over extended follow-up periods. This exploratory analysis will help determine the long-term potential of MBTs as therapeutic interventions. Lastly, this study will explore the psychosocial benefits associated with these therapies, such as improvements in quality of life and psychological well-being.

### Hypotheses

On the basis of the current understanding of MBTs, their effects on immune function, as well as the substantial gaps identified in the literature, this systematic review and meta-analysis aims to test several key hypotheses.

#### Primary hypothesis on the effectiveness of MBT in immune modulation

1a: MBTs contribute to the reduction of proinflammatory markers in children and adolescents. We hypothesize that MBTs such as yoga, meditation, and acupuncture lead to a significant reduction in the levels of proinflammatory markers such as TNF-α, IL-6, CXCL8, CRP, and serum amyloid A.

1b: MBTs increase the levels of anti-inflammatory markers in children and adolescents. We hypothesize that MBTs lead to a significant increase in the levels of anti-inflammatory markers such as IL-10 and TGF-β, which play a role in reducing inflammation and promoting immune regulation.

1c: MBTs modulate dual-function immunological markers. In particular, we hypothesize that MBTs lead to positive changes in growth factors such as vascular endothelial growth factor (VEGF), immune cell populations (e.g., T cells, B cells, NK cells, monocytes, and mast cells), antibodies (e.g., IgA, IgG, and IgM), and other markers such as ferritin, fibrinogen, and the ESR. These changes are expected to reflect an overall improvement in the balance and function of the immune system.

#### Secondary hypotheses on differential effects in subgroups

2a: The effects of MBTs differ between studies that include only children and adolescents and those that include a mix of children, adolescents, and adults. We hypothesize that MBTs will show more pronounced benefits in studies focusing exclusively on the younger population because of the specific physiological and psychological characteristics of children and adolescents and the more homogeneous study population.

2b: The effectiveness of MBTs differs between healthy children and adolescents and those with immune impairments. We hypothesize that individuals with immunological impairments will show more marked improvements in immune markers than healthy individuals due to their more fragile baseline level of immune response.

#### Exploratory hypotheses

##### Safety and tolerability of MBT in pediatric populations

MBTs are safe for use in children and adolescents, and few adverse effects have been reported. We hypothesize that MBTs have a low incidence of adverse events (e.g., less than 5% of participants reporting any significant discomfort) and high tolerability as indicated by a low dropout rate (less than 10%) across studies. These outcomes will be assessed within the study period and across the included interventions. Given the non-invasive nature of MBTs and their emphasis on relaxation, we expect their tolerability to remain consistent across different pediatric age groups and settings. To substantiate these claims, we focus on measurable outcomes, such as adverse event rates and dropout rates, rather than relying solely on the assumption of tolerability.

##### Longitudinal effects of MBT

The benefits of MBT on immune function are maintained over a longer period. We hypothesize that studies with longer follow-up periods, defined as at least 6 months postintervention, will show that the immunologically beneficial effects of MBTs on immune markers are maintained or even enhanced over time. This hypothesis addresses the potential for long-term benefits and lasting effects and the effects of MBTs on immune health.

##### Psychosocial benefits of MBT

MBTs improve the quality of life and psychological well-being of children and adolescents. We hypothesize that participants undergoing MBT will report significant improvements in quality of life and psychological well-being, reflecting the holistic benefits of these therapies beyond immunomodulation.

### Design

This research will be conducted as a systematic review and meta-analysis following the Preferred Reporting Items for Systematic Reviews and Meta-Analyses Protocols (PRISMA-P) guidelines [48]. For the systematic review, existing studies investigating the effectiveness and safety of MBTs in the modulation of immune markers in children and adolescents are identified, assessed, and summarized. If pooling of data is feasible, the meta-analysis will statistically summarize the results to provide a comprehensive and quantitative assessment of the findings. This review is registered with PROSPERO (Registration Number: CRD42024546585).

### Criteria for considering studies

The review will include published empirical studies investigating the associations between MBTs and immune markers in pediatric populations and studies with mixed cohorts, including both minors and adults. The studies considered will be randomized controlled trials (RCTs), non-RCTs, cohort studies, case–control studies, and single- and multi-arm studies with pre- and post-scores. Studies will be excluded if they do not provide empirical data on the associations between MBTs and immune markers.

#### Types of studies

The review will include the following:

- ◦ RCTs: studies in which participants are randomly assigned to an MBT intervention group or a control group.
- ◦ Non-RCTs: studies that use non-randomized methods to assign participants to an intervention or control group.
- ◦ Cohort studies: longitudinal studies that follow a group of participants over time to observe the effects of MBT on immune markers.
- ◦ Case-control studies: studies in which participants with a specific disease (e.g., HIV infection) are compared with participants without this disease to investigate their exposure to MBTs.
- ◦ Single-arm and multi-arm trials with pre- and post-scores: eligible studies must measure immune markers both before and after the MBT intervention, allowing for the assessment of within-group changes and, where applicable, between-group comparisons.

#### Types of populations

This systematic review focuses on children and adolescents under 21 years of age. This means that our search strategies and analyses will integrate both studies consisting exclusively of children and adolescents and mixed populations that include adults as well as minors. To ensure comprehensive coverage and precision in our study search, we will use the search strategy from Boluyte et al. [49], which compares different filters and strategies in terms of precision, and we will adopt the one with the highest efficiency to identify relevant studies with pediatric populations.

#### Exposure measures

The effectiveness of participation in MBTs will be analyzed. MBTs considered in this review include acupuncture, Healing Touch, hypnosis, hypnotherapy, massage therapy, meditation (including transcendental meditation and mindfulness), mantram (repetition of mantras), continuous passive movement therapy, the Feldenkrais method, the Alexender technique, Pilates (including Pilates exercises, Pilated-based exercises, and Pilates training), Rolfing (structural integration), Trager psychophysical integration, relaxation techniques, breathing exercises, guided imagery, muscle relaxation, spinal manipulation, physiotherapy modalities, Tai Chi, Qi Gong, yoga, dance therapy, music therapy, and journaling. The MBT terms were originally derived from two older systematic reviews and meta-analyses [40, 50], and extended by current expert consultations to ensure the inclusion of all relevant therapies.

#### Outcome measures

The primary outcome measures are changes in immune markers from baseline (before therapy) to various follow-up periods, classified as short-term (until 6 months), medium-term (6 to 12 months), and long-term (12–24 months) postintervention. This classification will allow for analysis of the immediate and sustained effects of MBTs. These markers include *proinflammatory* cytokines such as TNF-α and IL-6, chemokines such as CXCL8, acute phase proteins such as CRP and serum amyloid A. *Anti-inflammatory* markers such as IL-10 and TGF-β will also be included. Additionally, *dual function* markers, such as growth factors such as VEGF, immune cell populations (T, B, NK, mast cells, and monocytes), antibodies (IgA, IgG, and IgM), and other markers, such as ferritin, fibrinogen, and ESR, will be analyzed.

Information on simple RNA expression specifically targeting immune regulatory genes, such as cytokine genes (e.g., IL6, TNFA, and IL10), chemokine genes (e.g., CXCL8), immune cell marker genes (e.g., CD4, CD8A, and CD19), and acute phase response genes (e.g., CRP and SAA1), will also be included. Other omics data will be excluded owing to their complexity and the current lack of studies linking these directly to MBTs in pediatric populations.

Cortisol and other stress hormones, such as adrenaline, noradrenaline, dehydroepiandrosterone (DHEA), growth hormones, insulin-like growth factor (IGF)−1, endorphins, and oxytocin, will be excluded from this review. These mediators are excluded because their effects on the immune system are non-specific and can vary greatly depending on multiple factors, making it difficult to accurately interpret the direct effects on immune function after MBTs.

#### Inclusion and exclusion criteria

A table detailing the inclusion and exclusion criteria is presented (Table 1).

**Table 1:** Inclusion and exclusion criteria

Criteria	Inclusion	Exclusion
Study design	RCTs, non-RCTs, cohort studies, case–control studies, single-arm and multi-arm trials	Studies without empirical data on MBTs and immune markers
Population	Children and adolescents under 21 years, mixed populations including minors	Adults only
Outcomes	Changes in immune markers, gene expression related to immune regulation	Cortisol and other stress hormones
Languages	All languages (translated to English using DeepL Pro)	None

*RCTs* randomized controlled trials, *MBTs* mind-body-therapies

### Search strategy and data extraction

#### Search strategy

We will implement a comprehensive and systematic three-step search strategy. The first step involves the development of a search strategy tailored to the research question and databases. To ensure precision and comprehensiveness, we will use the search strategy published in Boluyt et al. [49], which compared different filters and strategies for identifying studies with pediatric populations. The most precise method will be adopted for this systematic review and meta-analysis. In addition, our search terms will include various MBT terms adapted from two published systematic reviews and meta-analyses [40, 50], supplemented with additional terms to ensure completeness.

Next, several electronic databases, including MEDLINE (encompassing PubMed content) and Embase via Ovid, PsycINFO, and CINAHL via EBSCOhost, Web of Science, and the Cochrane Library, will be searched. Additionally, trial registries such as ClinicalTrials.gov and the WHO International Clinical Trials Registry Platform (ICTRP) will be searched to capture ongoing trials. The search will consider studies published since the start of the respective database up to the search time so that a comprehensive and up-to-date synthesis of the available evidence is guaranteed. The detailed search strategy is available in Supplementary material 1.

Lastly, we will use the citationchaser tool [51] to manually identify citations and references of key articles to identify additional relevant studies that may not be captured in the original database search.

To minimize language bias, we will use DeepL Pro (https://www.deepl.com/) to translate entire publications from non-English languages into English. Since German is the native language of the author team, German publications will be reviewed in their original language. We have chosen English because we believe that AI-based translation is more advanced than other languages.

#### Selection of studies

Initially, two independent reviewers (SS and GN/MS) will screen all titles and abstracts identified by the search for eligibility. Studies in which participants without age information are mentioned will be included in the next step for a detailed review to ensure that relevant studies with children and adolescents are not overlooked.

The full-text articles of potentially eligible studies will be obtained and reviewed in detail. This step ensures that studies fulfill the inclusion criteria in terms of age, intervention, and outcomes. The general rule for abstract and full-text screening is that discrepancies between two reviewers are resolved through discussion and, if necessary, consultation with a third reviewer (AKK).

#### Data extraction

Data extraction will be performed independently by two reviewers (SS and GN/AKK) via a standardized data extraction form. The extracted data will include the following information:

- ◦ Study characteristics: record identifier, authors, year of publication, country where the research was conducted, type of publication, study year, study design (e.g., RCT, cohort, case-control), and sampling procedures.
- ◦ Participant characteristics: age, sex, health status (e.g., healthy vs. inflammation), and other relevant demographics.
- ◦ Intervention details: type of MBT (e.g., yoga, meditation, acupuncture), duration, frequency, and specifics of the intervention. The intervention details will be extracted in accordance with the Template for Intervention Description and Replication (TIDieR) [52] checklist to ensure comprehensive reporting of the intervention components.
- ◦ Outcome measures: type of immune marker (e.g., proinflammatory cytokines such as TNF-α and IL-6; anti-inflammatory cytokines such as IL-10; chemokines such as CXCL8; growth factors such as VEGF and TGF-β; acute phase proteins such as CRP and serum amyloid A; immune cell populations such as T, B, NK, and mast cells as well as monocytes; antibodies such as IgA, IgG, and IgM; other immune markers such as ferritin, fibrinogen, and ESR); measurement time points; and gene expression information relevant to immune regulation (e.g., the cytokine genes IL6, TNFA, and IL10; the chemokine gene CXCL8; the immune cell marker genes CD4, CD8A, and CD19; and the acute phase response genes CRP and SAA1). For each outcome, means and standard deviations (SDs) will be extracted, including changes from baseline with the corresponding SDs where available, to facilitate meta-analysis.
- ◦ Adverse effects: any reported adverse effects associated with MBT interventions.
- ◦ Psychosocial measures: quality of life, psychological well-being, and other patient-reported outcomes. While we will include data from a variety of validated instruments that measure these constructs, we will prioritize well-established and widely used questionnaires such as the SF-36 for quality of life and the Beck Depression Inventory (BDI) for psychological well-being. When pooling data for meta-analysis, we will consider the comparability of different instruments to ensure the robustness and interpretability of the results.

The participants’ state of health is categorized according to their common pathological features (e.g., psychiatric disorders, dermatological disorders). A board-certified pediatrician (GN) and a psychologist (SS) independently from each other will carry out this categorization, thereby ensuring an accurate and comprehensive classification from a medical and psychological point of view. If discrepancies arise during this classification process, a third person (MS) with sufficient expertise is consulted to resolve them.

Duplicate records will be identified and removed via Rayyan software (https://www.rayyan.ai/) to manage systematic reviews [53]. Rayyan recognizes duplicates via a similarity index that considers, for example, the title, journal name, and authors. If the degree of similarity is above 95%, the records will be considered duplicates and removed. For publications with a lower degree of similarity, each record will be carefully compared, and a decision on the duplication status (SS) is made. Data from studies appearing in multiple publications will be thoroughly documented, but only the primary or most comprehensive set of results will be used to avoid double counting. If detailed data are not accessible via publication, efforts will be made to contact the authors. The authors will be contacted via the corresponding author three times in total. There will be a 2-week period between the first and second approaches. The last approach will then follow 4 weeks after initial contact.

Disagreements between reviewers (SS and AKK) during data extraction will be resolved by consensus and, if necessary, with the involvement of a third reviewer (MS) to ensure accuracy and reliability. This meticulous process is designed to maintain the highest standards of data quality and integrity to ensure that the final analysis is based on the most accurate and comprehensive data available.

## Study quality and critical appraisal

### Quality assessment

The quality of the studies included will be assessed via common and standardized instruments. For RCTs, we will use the Cochrane Risk of Bias Tool (RoB 1) [54], which evaluates key domains such as sequence generation, allocation concealment, blinding, incomplete outcome data, selective reporting, and other sources of bias. Non-RCTs will be assessed via the Risk of Bias in Non-randomized Studies of Interventions (ROBINS-I) [55] tool, which considers biases before, during, and after the intervention. For observational studies, such as cohort and case–control studies, we will use the Newcastle‒Ottawa Scale (NOS) [56, 57], which assesses the selection of study groups, comparability of groups, and determination of exposure or outcome. Risk of bias will be assessed at the study level, as biases related to study design, participant selection, blinding, and data handling influence all reported outcomes. To further enhance transparency, risk of bias assessments will be categorized by outcome type, specifically by immune marker classification (proinflammatory, anti-inflammatory, and dual-function markers). This approach ensures a consistent and interpretable evaluation of methodological quality while accounting for potential variations in outcome measurement within each study.

The quality assessment will be conducted by two independent reviewers (AKK and MS). To ensure consistency and reliability in the assessment process, we will calculate the interrater reliability via Cohen’s κ [58]. Any disagreements between reviewers will be resolved through discussion, and if consensus cannot be reached, a third reviewer (GN) will be consulted. The results of the quality assessment will be used to interpret the review findings and perform sensitivity analyses to ensure the robustness of our conclusions. Studies identified as having a high risk of bias or poor methodological quality will be flagged. If a study is deemed to be of particularly low quality, it may be excluded from the meta-analysis, depending on the degree of bias and its potential impact on the overall findings. The results of the quality assessment will be used to interpret the review results and perform sensitivity analyses to ensure the robustness of our conclusions.

### Analysis

#### Descriptive analysis

The analysis will focus on quantitative approaches to summarize the findings from the studies included. Descriptive statistics such as measures of central tendency and measures of dispersion will be used to summarize the characteristics of the studies, participants, interventions, and outcomes. These statistical summaries will then provide a comprehensive overview of the study designs, sample sizes, participant demographics, types of MBT interventions, immune markers, and disease patterns measured. In addition to quantitative summaries, we will summarize the main findings of the studies included, highlighting patterns, trends, and any notable variations across studies. SS will conduct this analysis and all further analyses in close collaboration with AKK and RR. All authors will interpret the results.

#### Meta-analysis

For the meta-analysis, we will use specialized R software packages, including “meta”, “metafor”, and “robumeta”. The “meta” package will be used for general meta-analytic procedures, whereas “metafor” will allow for more advanced modeling, including meta-regression, and handling of more complex random effects models. The “robumeta” package may be used for robust variance estimation. The primary effect measures will include standardized mean differences (SMDs) for continuous outcomes, along with their 95% CIs. The SMD will be calculated via Hedges’ *g*, which corrects for small sample bias. For all meta-analyses, random effects models using restricted maximum likelihood (REML) [59] will be used as the primary approach to synthesize effect sizes because it is particularly appropriate given the anticipated variability across studies in terms of participant characteristics, intervention types, and outcome measures. To facilitate interpretation, Hedges’ *g* will be categorized using conventional benchmarks, where small effects correspond to *g* = 0.2, moderate effects to *g* = 0.5, and large effects to *g* ≥ 0.8. This approach accounts for both within-study and between-study variability, providing more generalizable results. To address potential differences in effect sizes across study designs, meta-regressions will be conducted with study design (RCT, non-RCT, observational) as a categorical moderator variable, provided that at least five studies per moderator category are available. This will allow us to evaluate whether study design significantly influences the observed effect sizes and, if so, quantify these differences. If the minimum number of studies per category is not reached, we will perform stratified meta-analyses, analyzing RCTs, non-RCTs, and observational studies separately. This ensures that studies with inherently different methodological qualities are not pooled together in a way that could distort results. Statistical significance will be judged based on *p* < 0.05*.* Given the extensive number of subgroup analyses planned in this review, there is an increased risk of Type I errors due to multiple testing. To address this, we will apply a false discovery rate (FDR) correction via the Benjamini‒Hochberg procedure to adjust *p*-values from subgroups and meta-regressions, prioritizing the control of false positives while preserving statistical power [60]. This method will help control the expected proportion of incorrectly rejected null hypotheses (false positives), ensuring that our findings remain robust despite the multiple comparisons. For cases in which meta-analyses are not adequate approaches for data synthesis, we will use a narrative synthesis of the reviewed article on the effects of the interventions.

Separate meta-analyses will be conducted for each class of immune markers: (1) proinflammatory markers, (2) anti-inflammatory markers, and (3) dual-function markers. If sufficient studies (at least three) are available for individual markers such as TNF-α or IL-6, separate meta-analyses are also performed for these individual markers. If only two studies are available, calculations could be carried out but would be rather unreliable because of the risk of highly distorted results due to opposing extreme values. By specifying the minimum number of three studies, we increase the precision of the estimate of the overall effect and reduce the risk that individual studies with extremely large effects disproportionately influence the overall estimate.

#### Assessing heterogeneity

The heterogeneity of the studies will be assessed via several statistical measures to ensure a comprehensive understanding of the variability in effect sizes. We will use the *I*^2^ statistic to quantify the proportion of total variation across studies that is due to heterogeneity rather than chance. *I*^2^ values of 25%, 50%, and 75% represent low, moderate, and high heterogeneity, respectively [61]. In addition, we will use the tau-squared (τ^2^) statistic, which estimates the between-study variance in effect sizes beyond what would be expected by chance alone. For this meta-analysis, we will use the REML method to estimate τ^2^, as it is widely regarded for its balance between bias and efficiency [59]. This measure provides insights into the extent of heterogeneity that cannot be attributed to sampling error. Furthermore, we will report the *Q* statistic that is sensitive to the number of studies included; therefore, we will interpret it alongside *I*^2^ and τ^2^ [62].

To provide a more nuanced understanding of the variability, we will also calculate prediction intervals [63]. If substantial heterogeneity is detected (e.g., *I*^2^ > 75%), we will consider potential causes of heterogeneity through subgroup analyses and meta-regression.

#### Subgroup analyses

In this systematic review and meta-analysis, subgroup analyses will be conducted with the following aims:

1. Studies with exclusively pediatric populations versus mixed populations (see “Secondary hypothesis on differential effects in subgroups” section).
2. Healthy individuals compared with individuals with immunological impairments (see “Secondary hypothesis on differential effects in subgroups”).
3. The long-term effects of MBT according to analyses of studies with follow-up periods (see “Exploratory hypothesis on longitudinal effects of MBT” section).

These analyses will provide information on whether the effectiveness of MBT varies according to age, health status, and duration of follow-up.

#### Sensitivity analyses

Sensitivity analyses will be conducted to assess the robustness and reliability of our results by systematically excluding studies with a high risk of bias or other methodological weaknesses. The exclusion is based on our quality assessment tools and allows us to determine whether overall results were significantly influenced by biased or methodologically weak studies. In addition, we will conduct separate analyses for high-quality versus low-quality studies. This comparison allows us to verify the consistency of the results across different quality levels.

One of our hypotheses is also a sensitivity analysis, as we are interested in whether high heterogeneity in the age of the study participants is an essential influence on the results of this systematic review and meta-analysis (see “Hypothesis on differential effects in subgroups”).

#### Assessment of publication bias

To assess the risk of publication bias, we will use several methods. First, we will create funnel plots to visually inspect the results for any asymmetry that may indicate publication bias. As part of this assessment, we will also formally test for funnel plot asymmetry via Egger’s test for weighted regression [64]. We will also calculate a failsafe N [65], which estimates the number of unpublished studies with null results that would be needed to change the results of the meta-analysis to non-significant. While the failsafe N provides a basic measure of robustness, it has been criticized for potentially overstating the stability of results in large meta-analyses [66]. Therefore, we will interpret this measure cautiously and supplement it with more recent methods.

Recognizing the limitations of the failsafe N, we will also consider using more advanced techniques such as *p*-curve analysis [67] or selection models [68], which offer deeper insights into the presence of publication bias by focusing on the distribution of p values or modeling the selection process of studies, respectively. If publication bias is detected in the first step, we will apply Duval and Tweedie’s trim-and-fill method [69, 70] in the next step to adjust for the given bias and obtain an estimate of the effect size after accounting for missing studies.

## Discussion

### Importance of the topic and potential impact of the review

The investigation of MBTs and their potential to modulate immune markers in children and adolescents is a timely and critical area of research. Chronic inflammation in this population is linked to long-term health issues, including autoimmune diseases, cardiovascular conditions, and mental health disorders [1, 71–73]. The ability of MBTs to influence immune function could provide a non-invasive, holistic approach to managing these conditions, offering significant benefits in both clinical practice and public health [74].

This systematic review and meta-analysis aim to synthesize the existing evidence on the effectiveness and safety of MBTs in modulating immune markers in pediatric populations. The findings from this review could have far-reaching implications, providing the evidence base needed to integrate MBTs into pediatric healthcare guidelines. This integration could lead to more comprehensive, multidisciplinary care strategies that address both the physical and the psychological needs of children and adolescents. Additionally, by identifying gaps in the current literature, this review could direct future research efforts toward the most promising areas, ultimately contributing to better health outcomes for young patients.

### Anticipated challenges and limitations

Several challenges are anticipated in conducting this systematic review and meta-analysis. One significant challenge is the expected heterogeneity across studies. MBTs encompass a wide range of interventions, and the included studies may vary greatly in their design, population characteristics, and outcome measures. This heterogeneity could complicate data synthesis and interpretation, particularly if the variability between studies is substantial. Additionally, differences in study design (e.g., RCTs vs. non-RCTs) present a potential limitation. While meta-regression and subgroup analyses will help address covariate shifts between these designs, the inherent differences in study quality and internal validity may influence the pooled results. To mitigate this, we will interpret findings from combined analyses with caution and provide design-specific effect size estimates where necessary. Publication bias is another concern. Despite efforts to include non-English studies and utilize advanced methods such as *p*-curve analysis and selection models, there remains the possibility that unpublished studies or those with null results could impact the findings. Additionally, the use of AI-based translation tools, while necessary to capture a global perspective, may introduce subtle inaccuracies, particularly in understanding cultural nuances or context-specific details.

### Justification for methodological choices

The methodological choices outlined in this protocol are designed to address these challenges and ensure a robust analysis. The use of the Cochrane RoB 1 and ROBINS-I tools for assessing study quality ensures a rigorous evaluation of the studies included, which is particularly important given the expected diversity in study designs. The decision to use the REML method for estimating τ^2^ in the context of random effects models is justified by its ability to balance bias and efficiency, especially when dealing with heterogeneity. The inclusion of non-English studies is crucial for capturing a comprehensive view of the global research on MBTs. This approach minimizes language bias and ensures that the review includes diverse perspectives, which is particularly important for MBTs that may have cultural or regional variations in practice.

### Ethical and practical considerations

Ethical considerations in this review include maintaining the confidentiality and integrity of data from all included studies. Although this review involves secondary data analysis and does not directly engage participants, it is essential to ensure that the data are handled responsibly and that the findings are reported transparently and without bias. Practically, the review process will require careful coordination among the research team. The dual-reviewer system for study selection and data extraction is intended to reduce subjective bias and ensure consistency, but it will require clear communication and an effective conflict resolution process to manage any discrepancies between reviewers.

### Dissemination

The results of this systematic review and meta-analysis will be disseminated through several channels to ensure broad reach and impact. The findings will be submitted to journals specializing in pediatric health, immunology, and integrative medicine. Presentations at national and international conferences will further disseminate the results, targeting audiences in pediatrics, complementary and integrative medicine, and public health. Additionally, fact sheets and summaries will be produced in collaboration with academic institutions and policy partners to inform clinical practice, guide future research, and support the development of evidence-based guidelines for integrating MBTs into pediatric healthcare.

## Conclusion

In conclusion, this protocol outlines a systematic and methodologically rigorous approach to reviewing the effects of MBTs on immune markers in children and adolescents. While challenges such as heterogeneity and publication bias are anticipated, the strategies outlined in this protocol are designed to mitigate these issues and ensure that the findings are robust, reliable, and clinically relevant. This review highlights that outcomes can significantly impact pediatric healthcare, offering new insights into the role of MBTs in managing immune-related conditions in young patients.

## Supplementary Information

Supplementary Material 1: Search strategy.
