# Supplementary material for: Mind–body therapies and their interplay with the immune system in children and adolescents: a protocol for a systematic review and meta-analysis
Source: Syst Rev. 2025 Apr 4;14:78. doi: 10.1186/s13643-025-02812-4 (PMC11969903; doi:10.1186/s13643-025-02812-4)
Supplement: Supplementary file 1 — Supplementary Material 1: Search strategy. [file 13643_2025_2812_MOESM1_ESM.pdf]

Supplementary file 1: Search Strategy

Ovid MEDLINE Search Strategy (adapted for other databases as needed)

Terms:

1 exp Infant/
2 exp Child/
3 exp Adolescent/
4 exp Pediatrics/
5 Infant*.ab,ti.
6 Newborn*.ab,ti.
7 Baby*.ab,ti.
8 Babies.ab,ti.
9 Neonat*.ab,ti.
10 Preterm*.ab,ti.
11 Prematur*.ab,ti.
12 Postmatur*.ab,ti.
13 Child*.ab,ti.
14 Adoles*.ab,ti.
15 Teen*.ab,ti.
16 Youth*.ab,ti.
17 Kid*.ab,ti.
18 Toddler*.ab,ti.
19 Pediatric*.ab,ti.
20 Paediatric*.ab,ti.
21 exp Infant/ **OR** exp Child/ **OR** exp Adolescent/ **OR** exp Pediatrics/ **OR** (Infant* or Newborn* **OR** Baby* **OR** Babies **OR** Neonat* **OR** Preterm* **OR** Prematur* **OR** Postmatur* **OR** Child* **OR** Adoles* **OR** Teen* **OR** Youth* **OR** Kid* **OR** Toddler* **OR** Pediatric* **OR** Paediatric*).ab,ti.
22 exp Mind-Body Therapies/
23 Mind-Body Therap*.ab,ti.
24 Acupunct*.ab,ti.
25 Hypnos*.ab,ti.
26 Hypnotherap*.ab,ti.
27 Massag*.ab,ti.
28 Meditat*.ab,ti.
29 Mindful*.ab,ti.
30 Mantram.ab,ti.
31 Relaxation Therap*.ab,ti.
32 Breathing Exercis*.ab,ti.
33 Feldenkrais.ab,ti.
34 Pilates.ab,ti.
35 Rolfing.ab,ti.
36 Trager psychophysical integration.ab,ti.
37 Guided Imagery.ab,ti.
38 Yoga.ab,ti.
39 Tai Ji.ab,ti.
40 Tai Chi.ab,ti.
41 Qi Gong.ab,ti.
42 Journaling.ab,ti.
43 Qigong.ab,ti.
44 Biofeedback.ab,ti.
45 Art Therap*.ab,ti.

46 Dance Therap*.ab,ti.

47 Autogenic Training.ab,ti.

48 Therapeutic Touch.ab,ti.

49 exp Mind-Body Therapies/ **OR** (Mind-Body Therap* **OR** Acupunct* **OR** Hypnos* **OR** Hypnotherap* **OR** Massag* **OR** Meditat* **OR** Mindful* **OR** Mantram **OR** Relaxation Therap* **OR** Breathing Exercis* **OR** Feldenkrais **OR** Pilates **OR** Rolfing **OR** Trager psychophysical integration **OR** Guided Imagery **OR** Yoga **OR** Tai Ji **OR** Tai Chi **OR** Qi Gong **OR** Journaling **OR** Qigong **OR** Biofeedback **OR** Art Therap* **OR** Dance Therap* **OR** Autogenic Training **OR** Therapeutic Touch).ab,ti.

50 exp Immune System/

51 Immune System.ab,ti.

52 Immun*.ab,ti.

53 Interleukin*.ab,ti.

54 Interferon*.ab,ti.

55 IFN*.ab,ti.

56 C-Reactive Protein.ab,ti.

57 CRP.ab,ti.

58 Tumor Necrosis Factor.ab,ti.

59 TNF.ab,ti.

60 Cytokin*.ab,ti.

61 Chemokin*.ab,ti.

62 Inflamm*.ab,ti.

63 Leukocyt*.ab,ti.

64 Lymphocyt*.ab,ti.

65 Monocyt*.ab,ti.

66 Macrophage*.ab,ti.

67 Natural Killer Cell*.ab,ti.

68 Ferritin*.ab,ti.

69 Fibrinogen*.ab,ti.

70 Blood Sedimentation.ab,ti.

71 ESR.ab,ti.

72 Growth Factor*.ab,ti.

73 Acute Phase Protein*.ab,ti.

74 Immune Cell*.ab,ti.

75 Antibod*.ab,ti.

76 Marker*.ab,ti.

77 Gene Expression.ab,ti.

78 exp Immune System/ **OR** (Immune System **OR** Immun* **OR** Interleukin* **OR** Interferon* **OR** IFN* **OR** C-Reactive Protein **OR** CRP **OR** Tumor Necrosis Factor **OR** TNF **OR** Cytokin* **OR** Chemokin* **OR** Inflamm* **OR** Leukocyt* **OR** Lymphocyt* **OR** Monocyt* **OR** Macrophage* **OR** Natural Killer Cell* **OR** Ferritin* **OR** Fibrinogen* **OR** Blood Sedimentation **OR** ESR **OR** Growth Factor* **OR** Acute Phase Protein* **OR** Immune Cell* **OR** Antibod* **OR** Marker* **OR** Gene Expression).ab,ti.

79 21 AND 49 AND 78
